# Phenolic-rich fraction of *Solanum betaceum* mitigates atorvastatin-induced myotoxicity through antioxidant mechanisms in female wistar rats

**DOI:** 10.1016/j.toxrep.2025.102049

**Published:** 2025-05-16

**Authors:** Rachael Jackie Mpumbya, Josiah Eseoghene Ifie, Ayomide Victor Atoki, Stella Maris Kembabazi, Solomon Adoni Mbina, Eliah Kwizera, Gilbert Akankwasa, Mary Gorret Ablinda, Fred Bwamble, Siida Robert, Pastori Mujinya, Andrew Kisakye, Ilemobayo Victor Fasogbon, Nancy B. Mitaki, Ibrahim Babangida Abubakar, Daniel Ejike Eze, Nwokike Matthew Onyemaechi, Sana Noreen, Patrick Maduabuchi Aja

**Affiliations:** a Department of Biochemistry, Faculty of Biomedical Sciences, Kampala International University, Uganda; b Department of Physiology, School of Medicine, Kabale University, Kabale, Uganda; c Department of Pharmacology, FINS Medical University, Fort Portal, Uganda; d Department of Medical Laboratory Science, School of Allied Health Sciences, Kampala International University, Uganda; e University Institute of Diet and Nutritional Sciences, The University of Lahore, Lahore, Pakistan

**Keywords:** *Solanum betaceum*, Atorvastatin, Myotoxicity, Antioxidant, Muscle enzymes, Statin induced myopathy

## Abstract

**Background:**

Atorvastatin, a widely prescribed cholesterol-lowering medication, has been linked to statin-induced myotoxicity, a condition marked by elevated muscle enzymes and oxidative stress. While statins are essential for managing hypercholesterolemia and preventing cardiovascular diseases, their myotoxic side effects limit broader clinical use. This study investigated the myo-protective effects of the phenolic-rich fraction of *Solanum betaceum* (PRFSB) in mitigating atorvastatin-induced muscle damage in female Wistar rats.

**Methods:**

Thirty female Wistar rats were divided into five groups: (1) a true control group receiving distilled water, (2) an atorvastatin-only group, and three treatment groups receiving PRFSB at varying doses (100, 200, and 400 mg/kg) alongside atorvastatin. Muscle enzymes (creatine kinase and lactate dehydrogenase), oxidative stress markers (catalase, superoxide dismutase, and malondialdehyde), and histopathological changes were assessed.

**Results:**

Atorvastatin significantly elevated serum CK, LDH and oxidative stress markers, while PRFSB administration, particularly at 400 mg/kg, significantly reduced these elevations (p < 0.05). PRFSB restored muscle enzyme levels, normalized antioxidant defenses and reduced lipid peroxidation. Histological analysis revealed that PRFSB-treated groups exhibited preserved muscle architecture with minimal inflammation.

**Conclusion:**

PRFSB effectively alleviated atorvastatin-induced myotoxicity by reducing oxidative stress, restoring muscle biomarkers and protecting tissue integrity. These findings suggest that PRFSB holds promise as an adjunct therapy to mitigate statin-induced muscle toxicity, warranting further exploration in clinical settings.

## Highlights

• Solanum betaceum's phenolic-rich fraction (PRFSB) reduced muscle damage from atorvastatin by lowering creatine kinase and lactate dehydrogenase levels.
• PRFSB normalized levels of antioxidant enzymes catalase and superoxide dismutase, highlighting its potential in counteracting oxidative stress induced by atorvastatin.
• PRFSB reduced malondialdehyde levels dose-dependently, protecting against lipid peroxidation and cell damage.
• PRFSB-treated groups showed near-normal muscle architecture with reduced inflammation, unlike atorvastatin-only group.
• PRFSB shows promise as a natural therapy to mitigate statin-induced muscle toxicity.

## Graphical Abstract

**Figure:**
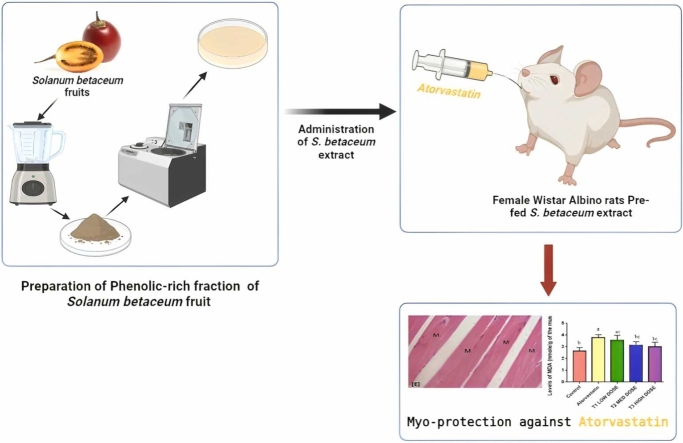

## 1 Introduction

Statins, particularly atorvastatin, are among the most commonly prescribed lipid-lowering agents used to reduce cholesterol levels and prevent cardiovascular diseases [8]. Despite their proven efficacy, long-term statin therapy is associated with various adverse effects, including hepatotoxicity, nephrotoxicity and most notably, statin-induced myotoxicity (SIM) [7]. Myotoxicity encompasses a spectrum of muscle-related disorders ranging from mild myalgia to severe rhabdomyolysis, characterized by elevated serum levels of muscle damage biomarkers such as creatine kinase (CK), lactate dehydrogenase (LDH) and alanine aminotransferase (ALT) [14]. The pathophysiology of SIM remains complex and multifactorial, with oxidative stress, mitochondrial dysfunction and disruption of calcium homeostasis playing central roles [6]. Given these mechanisms, there is an increasing interest in the use of natural antioxidants as potential protective agents against statin-associated muscle toxicity.

A growing body of evidence suggests that oxidative stress is a critical mediator of statin-induced muscle damage [12]. Statins are known to impair mitochondrial function by inhibiting coenzyme Q10 biosynthesis, leading to reduced ATP production, increased reactive oxygen species (ROS) generation and subsequent oxidative damage to muscle tissues [7]. This oxidative imbalance disrupts muscle homeostasis, exacerbating protein degradation and inflammatory responses, which further contribute to muscle dysfunction [20]. Consequently, antioxidant-based interventions have been explored to counteract these effects, with some studies highlighting the protective potential of phytochemicals in preserving mitochondrial integrity and muscle function [31].

*Solanum betaceum* (tree tomato or tamarillo) is a fruit known for its rich composition of phenolic compounds, including flavonoids, phenolic acids and furanones, which possess strong antioxidant and anti-inflammatory properties [29]. Previous studies have demonstrated the health benefits of *S. betaceum*, particularly in the modulation of oxidative stress and inflammation in various disease models [1]. However, its potential protective role against SIM has not been thoroughly investigated. The choice of female Wistar rats is based on their distinct hormonal profile, which influences oxidative stress responses and muscle metabolism. Estrogen has been shown to modulate mitochondrial biogenesis and antioxidant defense mechanisms, making female models relevant for assessing PRFSB’s efficacy in mitigating SIM. Given the phytochemical profile of *S. betaceum*, it is hypothesized that its phenolic-rich fraction (PRFSB) may mitigate atorvastatin-induced muscle damage by enhancing endogenous antioxidant defense mechanisms, reducing oxidative damage and modulating mitochondrial function.

Several bioactive compounds present in PRFSB have been associated with protective effects against oxidative stress-induced cellular damage. For instance, flavonoids such as quercetin and kaempferol, commonly found in *S. betaceum*, have been shown to improve mitochondrial function and reduce lipid peroxidation in muscle tissues. It is important to consider that some of these phytochemicals, particularly flavonoids and phenolic acids, may exhibit weak phytoestrogen-like activity [25]. As such, these compounds could potentially act as endocrine disruptors, influencing the hormonal profile of female subjects, especially considering the hormonal cyclicity of females. Such changes could potentially affect basal metabolism, warranting further investigation into their endocrine-modulating effects. Furthermore, phenolic acids such as chlorogenic acid have been reported to enhance enzymatic antioxidant activity, including superoxide dismutase (SOD) and catalase (CAT), thereby neutralizing ROS and preventing oxidative damage [23]. The potential synergistic effects of these compounds could contribute to the attenuation of atorvastatin-induced myotoxicity.

The present study aims to evaluate the protective effects of PRFSB against atorvastatin-induced myotoxicity in female Wistar rats. The choice of female rats is justified by their distinct hormonal profile, which influences oxidative stress responses and muscle metabolism. Estrogen has been shown to modulate mitochondrial biogenesis and antioxidant defense mechanisms, making female models relevant for assessing PRFSB’s efficacy in mitigating SIM [26].

## 2 Materials and methods

### 2.1 Chemicals

All chemicals utilized in this study were procured from Sigma Aldrich (St. Louis, MO, USA), unless stated otherwise.

### 2.2 Preparation of the phenolic-rich fraction from *Solanum betaceum* fruit extract

Fresh red *Solanum betaceum* (Tamarillo) fruits were sourced from the Basajja market in Bushenyi district and stored at 4°C in the Biochemistry laboratory at the Faculty of Biomedical Sciences, Kampala International University, Western Campus. The fruits were thoroughly cleaned with tap water to remove any debris or soil. After cleaning, the fruit samples were chopped into small pieces and ground using an electric blender or grinder. A total of 5000 g of the ground tamarillo fruit paste was extracted with 10 L of 50 % ethanol in a sealed bottle at room temperature for 72 hours, with intermittent stirring. The mixture was then filtered through muslin cloth, and the ethanol extract was allowed to air-dry for 24 hours. The dried crude extract was subjected to acetone partitioning to isolate the phenolic-rich fraction, following the method described by Rohilla and Mahanta [19]. This phenolic-rich fraction was subsequently analyzed using gas chromatography-mass spectrometry (GC-MS), as outlined by Singh and Chaturvedi [22], and utilized for *in-vivo* studies.

### 2.3 Acute toxicity test determination

In this study, the acute toxicity was evaluated using the limit dose method, following the up-and-down approach. To begin, one female rat was fasted overnight and then given an oral dose of 2000 mg/kg of the extract. The rat was observed closely for the first 4 hours after administration and then continuously monitored for the next 24 hours. Daily observations were carried out for a period of 14 days to check for any signs of toxicity. Since the first rat survived without any noticeable physical or behavioural changes, four more female rats were administered the same dose after fasting for 4 hours. These rats were also carefully monitored for signs of toxicity over a 14-day period [17], [2], [21]. At the 2000 mg/kg limit dose, none of the rats displayed any abnormal physical or behavioural symptoms during the initial 24 hours or throughout the 14-day observation period. Consequently, lower doses were selected for further testing, in accordance with OECD guideline No. 425. The doses chosen were 10 % of the maximum dose (200 mg/kg) for the intermediate dose, half of this intermediate dose (100 mg/kg) for the low dose, and twice the intermediate dose (400 mg/kg) for the high dose. These dose selections were based on the lack of toxic effects at the limit dose, as per OECD recommendations [17].

### 2.4 Animals

A total of 35 female Wistar albino rats, each approximately 12 weeks old, were used in this study. Five of these rats were designated for the determination of the LD50, while the remaining 30 were used in the experimental phase. The animals were sourced from the Animal Facility at Kampala International University's Western Campus in Uganda. For housing, six plastic cages with dimensions of 16 × 9 in. (144 square inches in total) were used to accommodate the rats. The cages were kept in a well-ventilated animal facility under controlled conditions, including a 12-hour light/dark cycle and regulated room temperature. Before the study began, the rats were allowed to acclimate to the laboratory environment for a period of seven days. Ethical approval for the study was granted by the Institutional Experimental Animal Welfare and Ethics Committee, with the reference number BSU-REC-2023–167, and all experimental procedures adhered to the approved guidelines.

### 2.5 Experimental grouping

The thirty rats were randomly divided into five groups, with each group containing six animals. The sample size was calculated using the formula E = total number of animals - total number of groups, where E represents the degrees of freedom for the analysis of variance (ANOVA). In this case, E = 30–5 = 25. According to standard guidelines, the E value should ideally fall between 10 and 20. If E is less than 10, increasing the number of animals could improve the significance of the results, while an E value greater than 20 suggests that adding more animals would not enhance the statistical power of the findings.

The treatment groups were organized as follows:

**Group 1** (control) received distilled water.

**Group 2** was given atorvastatin at a dose of 7.089 mg/kg body weight (b.w.).

**Group 3** received 100 mg/kg b.w. of *Solanum betaceum* extract, administered 90 minutes before the atorvastatin dose of 7.089 mg/kg b.w.

**Group 4** was treated with 200 mg/kg b.w. of *S. betaceum* extract, also given 90 minutes prior to the same dose of atorvastatin.

**Group 5** received 400 mg/kg b.w. of *S. betaceum* extract, 90 minutes before the atorvastatin dose.

All treatments were administered daily for a period of 28 days.

### 2.6 Assessment of biochemical parameters

Following a 28-day experimental period, rats were fasted overnight and then euthanized by decapitation under halothane anesthesia. Blood samples were collected via cardiac puncture and placed in non-heparinized vacutainers to allow clotting. After one hour, serum was separated by centrifugation at 3000 x g for 5 minutes, using a Pasteur pipette to extract it. The serum was analyzed for creatine kinase (CK) and lactate dehydrogenase (LDH) activity. Muscle tissue was excised, rinsed with ice-cold 1.15 % KCl solution, blotted, and weighed. The tissue was then homogenized in 0.1 M phosphate-buffered saline (PBS) at a ratio of 1:5 (w/v, pH 6.4), followed by centrifugation at 4000 x g for 20 minutes. The resulting supernatant was used to measure catalase (CAT), superoxide dismutase (SOD) and malondialdehyde (MDA) levels.

### 2.7 Histopathological analysis

For the histological examination, tissue preparation followed previously established protocols [24], [4]. Samples from skeletal muscle and liver were collected and preserved in 10 % formaldehyde for three days. After fixation, the tissues were processed and sectioned using an automated tissue processor and a rotary microtome. Sections were then stained with Hematoxylin and Eosin (H&E) for visualization, following standard histological procedures [11]. Images of the stained sections were captured using an Olympus BH2 light microscope, equipped with a Nikon Digital Sight DS-L1 camera (Nikon Corporation, Japan).

### 2.8 Statistical analysis

Data were expressed as mean values along with their standard errors (SEM). To assess the statistical differences, a one-way analysis of variance (ANOVA) was performed, followed by Tukey’s post hoc test for multiple comparisons. The analysis was conducted using GraphPad Prism 5 software (GraphPad Software, California, USA). A p-value of 0.05 or below was considered statistically significant. Prior to ANOVA, data normality was assessed using the Shapiro-Wilk test, and homogeneity of variance was evaluated using Levene’s test. Effect sizes (eta-squared, η²) were also calculated to provide a measure of the biological relevance of observed differences (Table. 3.1).

**Table 3.1:** Major GC-MS Constituents of the Phenolic-Rich Fraction of *Solanum betaceum*.

S/N	Retention Time (RT, min)	Compound Name	Molecular Formula	Concentration (%)
1	4.1908	2(5 H)-Furanone	C₄H₄O₂	0.50
2	4.2781	Cyclobutane, 1,2:3,4-di-O-ethylboranediyl-	C₈H₁₄B₂O₄	0.23
3	4.3516	Proline, 2-methyl−5-oxo-, methyl ester	C₇H₁₁NO₃	0.52
4	4.5639	2-Furancarboxaldehyde, 5-methyl-	C₆H₆O₂	3.44
5	5.3357	2,4(1 H,3 H)-Pyrimidinedione, 5-hydroxy-	C₄H₄N₂O₃	0.36
6	5.3613	4-t-Butoxy−3-hydroxy-butyric acid, ethyl ester	C₁₀H₂₀O₄	0.04
7	6.0727	4H-Pyran−4-one, 2,3-dihydro−3,5-dihydroxy−6-methyl-	C₆H₈O₄	16.32
8	6.706	Morpholine, TMS derivative	C₇H₁₇NOSi	0.21
9	6.7737	5-Hydroxymethylfurfural	C₆H₆O₃	16.45
10	11.5187	2-Thio−2,4-oxazolidinedione	C₃H₃NO₂S	0.46
11	22.9931	*n*-Hexadecanoic acid	C₁₆H₃₂O₂	1.88
12	29.0056	Octadecanoic acid	C₁₈H₃₆O₂	0.86
13	36.8414	1,3-Benzenedicarboxylic acid, bis(2-ethylhexyl) ester	C₂₄H₃₈O₄	2.63

## 3 Results

### 3.1 Effect of PRFSB on serum creatine kinase and lactate dehydrogenase levels

Administration of atorvastatin (7.089 mg/kg body weight) significantly elevated serum creatine kinase (CK) and lactate dehydrogenase (LDH) levels, indicating marked muscle damage (p < 0.05) when compared to the control group. CK and LDH levels were observed to be significantly elevated in the atorvastatin-only group, consistent with myotoxic effects of the drug. Treatment with PRFSB at doses of 100 mg/kg, 200 mg/kg, and 400 mg/kg significantly attenuated these elevations (p < 0.05). The 200 mg/kg dose of PRFSB produced the greatest reduction in CK and LDH levels, restoring them nearly to baseline values, followed closely by the 400 mg/kg dose (Fig. 1a, Fig. 1b). This suggests a dose-dependent myo-protective effect of PRFSB against atorvastatin-induced muscle injury.

**Fig. 1a:**
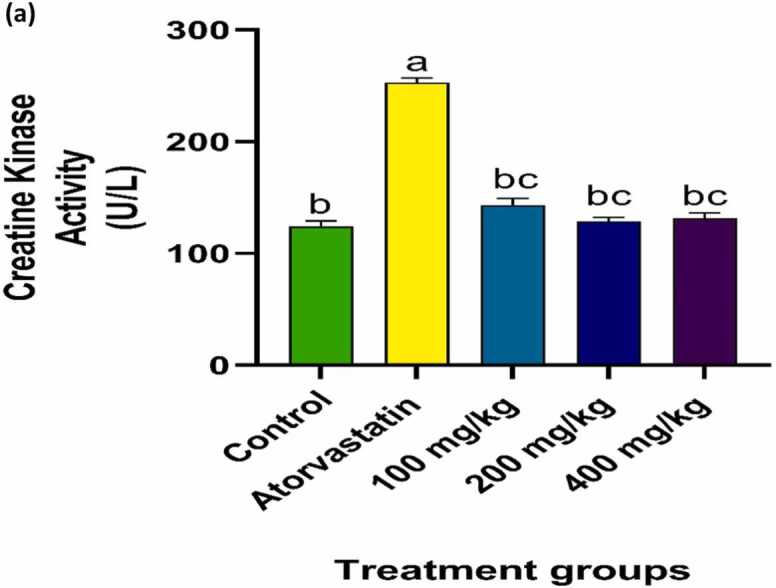
Effect of PRFSB on serum creatine kinase activity in atorvastatin-induced myotoxicity in Wistar rats. Data are shown as mean ± S.D (n = 6). Mean values with different superscripts (a, b, c) are significantly different at p < 0.05. a. indicates statistically significant difference compared to the control group. b indicates statistically significant difference compared to the atorvastatin-only group. c indicates statistically significant difference between PRFSB-treated groups at different doses.

**Fig. 1b:**
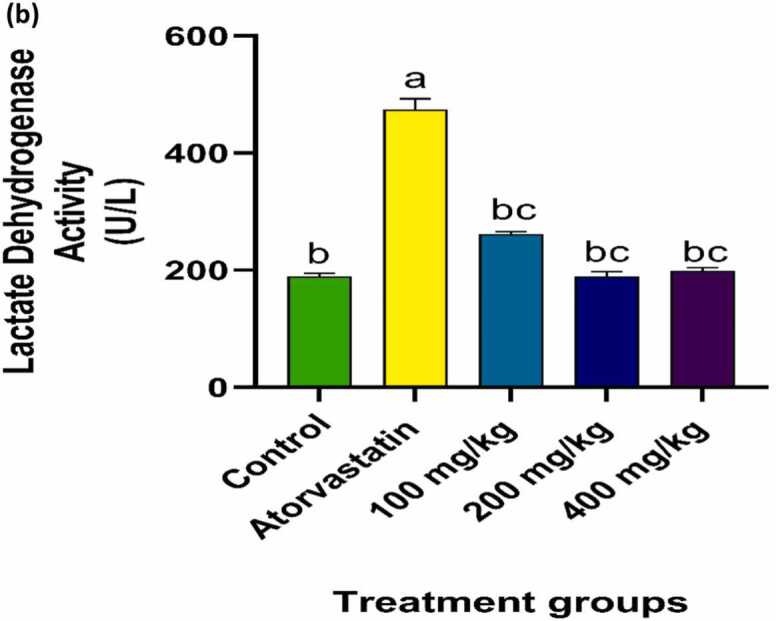
Effect of PRFSB on serum lactate dehydrogenase activity in atorvastatin-induced myotoxicity in Wistar rats. Data are shown as mean ± S.D (n = 6). Mean values with different superscripts (a, b, c) are significantly different at p < 0.05. a indicates statistically significant difference compared to the control group. b indicates statistically significant difference compared to the atorvastatin-only group. c indicates statistically significant difference between PRFSB-treated groups at different doses.

### 3.2 Effect of PRFSB on muscle antioxidant enzymes

Atorvastatin administration significantly disrupted muscle antioxidant defenses, as indicated by elevated catalase (CAT) and superoxide dismutase (SOD) activities (p < 0.05). Treatment with PRFSB significantly normalized these enzyme activities (p < 0.05) in a dose-dependent manner. For CAT activity, the 200 mg/kg dose exhibited the greatest normalization, while for SOD activity, the 400 mg/kg dose was most effective (Fig. 2a, Fig. 2b).

**Fig. 2a:**
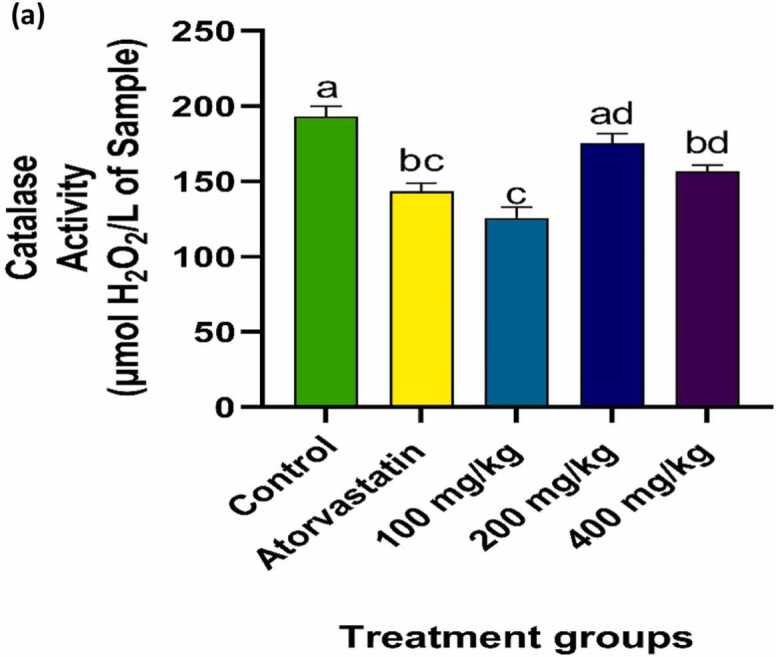
Effects of PRFSB on muscle catalase (CAT) activity in atorvastatin-induced myotoxicity in Wistar rats. Data are shown as mean ± S.D (n = 6). Mean values with different superscripts (a, b, c, d) are significantly different at p < 0.05. a indicates statistically significant difference compared to the control group. b indicates statistically significant difference compared to the atorvastatin-only group. c indicates statistically significant difference compared to the group treated with 100 mg/kg PRFSB. d indicates statistically significant difference compared to the group treated with 200 mg/kg PRFSB.

**Fig. 2b:**
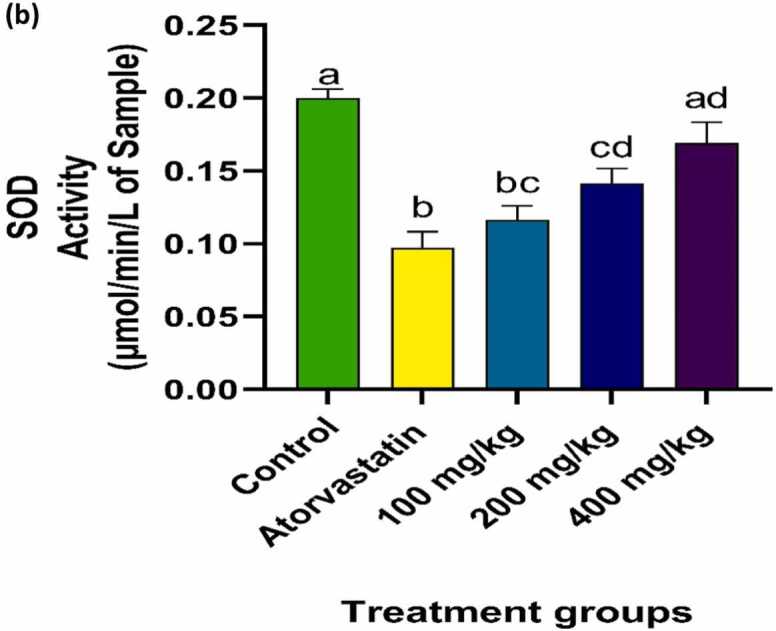
Effects of PRFSB on muscle superoxide dismutase (SOD) activity in atorvastatin-induced myotoxicity in Wistar rats. Data are shown as mean ± S.D (n = 6). Mean values with different superscripts (a, b, c, d) are significantly different at p < 0.05. a indicates statistically significant difference compared to the control group. b indicates statistically significant difference compared to the atorvastatin-only group. c indicates statistically significant difference compared to the 100 mg/kg PRFSB treatment group. d indicates statistically significant difference compared to the 200 mg/kg PRFSB treatment group.

### 3.3 Effect of PRFSB on muscle lipid peroxidation

Lipid peroxidation, as assessed by malondialdehyde (MDA) levels, was significantly elevated in the atorvastatin-treated group compared to controls (p < 0.05), indicating oxidative damage to muscle cell membranes. However, administration of PRFSB resulted in a significant dose-dependent reduction in MDA levels (p < 0.05), with the 400 mg/kg dose exhibiting the greatest reduction (Fig. 2c). This suggests that PRFSB mitigates lipid peroxidation and protects muscle membranes from oxidative damage induced by atorvastatin.

**Fig. 2c:**
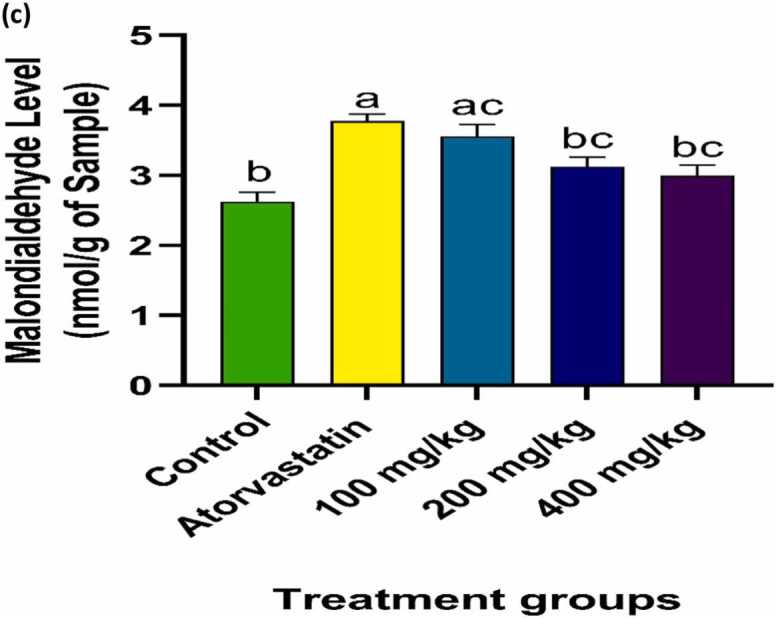
Effects of PRFSB on muscle malondialdehyde (MDA) levels in atorvastatin-induced myotoxicity in Wistar rats. Data are shown as mean ± S.D (n = 6). Mean values with different superscripts (a, b, c) are significantly different at p < 0.05. a indicates statistically significant difference compared to the control group. b indicates statistically significant difference compared to the atorvastatin-only group. c indicates statistically significant difference among PRFSB-treated groups at different doses.

### 3.4 Histopathological findings

**Fig. 3:**
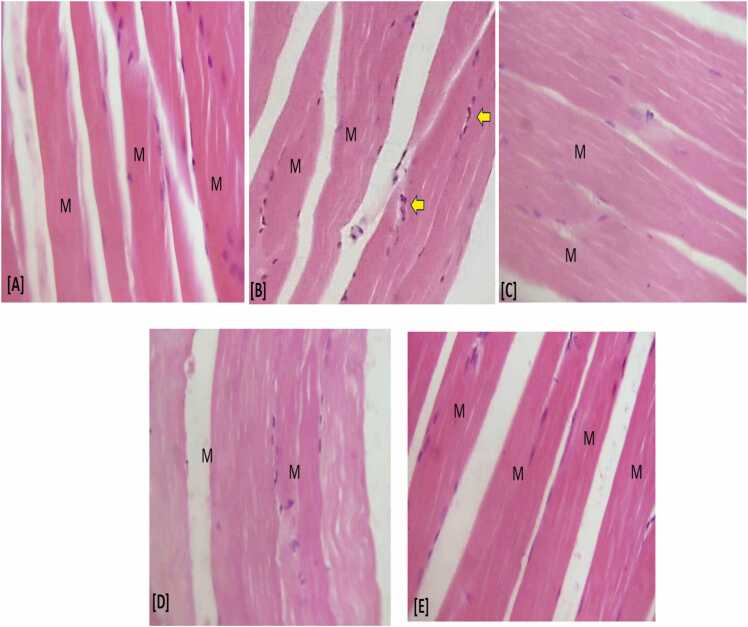
Photomicrograph of muscle tissues from the different experimental groups: (A): Showing the normal control group (B): Atorvastatin-treated group (C): Treatment group with *S. betaceum* extract at 100 mg/Kg (D): Treatment group with *S. betaceum* extract at 200 mg/Kg (E) Treatment group with *S. betaceum* at 400 mg/Kg The yellow arrow indicate inflammatory cells infiltration and M indicate the myocytes (H and E; x400).

Histological examination of muscle tissues was conducted by comparing tissue sections from all experimental groups to the control group as a baseline for normal tissue structure. A standard histological scoring method was used, which included qualitative evaluation of myocyte integrity, inflammatory cell infiltration and muscle fiber structure. Given that female rats exhibit distinct muscle metabolism due to the effects of estradiol, this was considered when selecting the experimental model, as these metabolic differences are an important aspect of the study's aims. The results presented reflect the histological evaluation based on these criteria (Fig. 3).

## 4 Discussion

The present study demonstrates the potent myo-protective effects of the phenolic-rich fraction of *Solanum betaceum* (PRFSB) against atorvastatin-induced myotoxicity in female Wistar rats. Statin-induced myopathy, a well-recognized side effect of long-term statin therapy, remains a significant clinical concern, limiting the broader therapeutic utility of drugs like atorvastatin. In this study, we investigated the potential protective effects of PRFSB on muscle damage, oxidative stress and histopathological alterations induced by atorvastatin. The results suggest that PRFSB effectively mitigates the myotoxic effects of atorvastatin, supporting its use as a potential adjunct therapy to reduce statin-associated muscle damage.

Statin-induced myotoxicity is characterized by elevated levels of muscle-derived enzymes, particularly creatine kinase (CK) and lactate dehydrogenase (LDH), which serve as biomarkers for muscle damage. In the current study, atorvastatin administration led to a significant increase in serum CK and LDH levels, indicating muscle cell injury, a finding that aligns with previous reports of statin-induced myopathy [15], [3]. The disruption of muscle cell integrity allows CK and LDH to leak into the bloodstream, reflecting underlying muscle degeneration [16].

The administration of PRFSB resulted in a significant reduction in CK and LDH levels, with the highest dose (400 mg/kg) almost completely reversing atorvastatin-induced elevations. This reduction suggests that PRFSB exerts a protective effect on muscle cells by preserving cell membrane integrity and preventing enzyme leakage. The phenolic compounds present in *Solanum betaceum* may have contributed to this protective effect by enhancing cell resilience and reducing the extent of muscle damage. These findings are consistent with other studies that have reported the protective effects of polyphenol-rich extracts in drug-induced myotoxicity [18].

Oxidative stress is a major contributor to statin-induced myopathy, as statins are known to impair mitochondrial function and generate excessive reactive oxygen species (ROS). ROS can damage muscle cells, leading to lipid peroxidation, protein oxidation and DNA damage [13]. In this study, atorvastatin administration was associated with elevated activities of catalase (CAT) and superoxide dismutase (SOD), suggesting a compensatory response to increased oxidative stress. These findings are consistent with the notion that atorvastatin induces oxidative stress in muscle tissues, as previously reported [9], [5].

Treatment with PRFSB significantly normalized CAT and SOD activities, particularly at the highest dose of 400 mg/kg, indicating that the extract can restore the balance of antioxidant defenses. This suggests that the phenolic compounds in PRFSB may enhance the activities of endogenous antioxidant enzymes, thereby reducing oxidative damage. Flavonoids and polyphenols, the primary constituents of PRFSB, have been shown to upregulate antioxidant defense mechanisms by directly scavenging ROS and stimulating the production of antioxidant enzymes [27]. By boosting antioxidant defenses, PRFSB may have helped to reduce oxidative stress and prevent the ensuing muscle damage observed in atorvastatin-treated rats.

Lipid peroxidation, a marker of oxidative damage to cell membranes, is a key feature of statin-induced muscle toxicity. In the current study, atorvastatin-treated rats exhibited significantly elevated levels of malondialdehyde (MDA), a byproduct of lipid peroxidation. These findings are consistent with previous reports that statins, including atorvastatin, increase oxidative damage in muscle tissues by promoting lipid peroxidation [13], [3]. Elevated MDA levels reflect the extent of oxidative damage to membrane lipids, which compromises cell membrane integrity and function.

Administration of PRFSB significantly reduced MDA levels in a dose-dependent manner, with the 400 mg/kg dose showing the most pronounced effect. This reduction in MDA levels suggests that PRFSB effectively inhibits lipid peroxidation, thereby protecting muscle cell membranes from oxidative damage. The ability of PRFSB to reduce lipid peroxidation may be attributed to its high polyphenol content, which has been shown to neutralize free radicals and prevent the propagation of oxidative chain reactions [10]. These results are further supported by the histopathological findings, which revealed a near-normal muscle architecture in PRFSB-treated rats, with minimal evidence of inflammation or degeneration.

Histological examination of muscle tissues provided additional insights into the protective effects of PRFSB. The atorvastatin-treated group exhibited marked myocyte degeneration and inflammatory cell infiltration, consistent with statin-induced myopathy. These pathological changes reflect the extensive muscle damage caused by oxidative stress and impaired cellular integrity. However, treatment with PRFSB significantly ameliorated these histological abnormalities, particularly at the highest dose of 400 mg/kg. The muscle tissues of rats treated with PRFSB showed minimal signs of degeneration and inflammation, supporting the conclusion that PRFSB offers substantial protection against atorvastatin-induced myotoxicity.

These findings suggest that PRFSB not only protects against biochemical alterations but also preserves muscle tissue architecture, further corroborating its myo-protective effects. Previous studies have highlighted the anti-inflammatory and antioxidant properties of polyphenols in preventing tissue damage, which may explain the observed histopathological improvements [28].

The protective effects of PRFSB observed in this study may be attributed to its rich content of phytochemicals. The GC-MS analysis of PRFSB revealed the presence of several bioactive compounds, including flavonoids, phenolic acids and organic acids, which are known for their antioxidant anti-inflammatory and cytoprotective properties. Key identified compounds such as 5-hydroxymethylfurfural, n-hexadecanoic acid and octadecanoic acid have been reported to modulate oxidative stress, lipid metabolism and inflammatory responses [30]. The presence of these bioactives suggests that PRFSB exerts its protective effects through multiple mechanisms, including scavenging of free radicals, inhibition of lipid peroxidation and modulation of inflammatory signaling pathways. These findings highlight the therapeutic potential of PRFSB in mitigating atorvastatin-induced toxicity and warrant further investigation into its specific molecular targets. One potential mechanism through which PRFSB exerts its protective effects is by enhancing the activities of endogenous antioxidant enzymes, such as CAT and SOD, thereby reducing ROS levels and preventing oxidative damage. Additionally, polyphenols are known to inhibit pro-inflammatory pathways, which may help reduce muscle inflammation and promote tissue repair [27].

Moreover, the ability of PRFSB to reduce lipid peroxidation suggests that it may help stabilize cell membranes and prevent further oxidative damage. By preserving membrane integrity and enhancing antioxidant defenses, PRFSB may protect muscle cells from the deleterious effects of atorvastatin, thus mitigating the severity of statin-induced myopathy.

Although the protective effects of PRFSB appear to be mediated primarily by its antioxidant properties, other potential mechanisms should be considered. Statins have been shown to impair calcium homeostasis in muscle cells, leading to increased susceptibility to muscle damage [20]. Given that polyphenols have been reported to modulate calcium signaling pathways, future studies would explore whether PRFSB exerts protective effects through calcium homeostasis regulation. Additionally, PRFSB may influence mitochondrial biogenesis and apoptotic pathways, as observed with other phytochemicals possessing antioxidant properties [31]. Further mechanistic studies focusing on mitochondrial DNA damage, cytochrome c release and caspase activation would provide deeper insights into PRFSB’s role in muscle protection.

The exclusive use of female Wistar rats in this study was based on their hormonal profile, which influences oxidative stress responses and muscle metabolism [26]. Estrogen has been shown to exert protective effects against oxidative damage by enhancing mitochondrial function and upregulating antioxidant defenses. While this study provides valuable insights, future research would investigate the effects of PRFSB in male rats to determine whether sex-based differences exist in its protective efficacy. Given that male rats exhibit different oxidative stress and inflammatory responses to statins, such studies would be essential for broader translational relevance.

One limitation of this study is the absence of a PRFSB-only control group, which would have allowed for the differentiation of its basal effects from its protective role against atorvastatin toxicity. Additionally, while this study focused on enzymatic antioxidants, other oxidative stress markers, such as reduced glutathione (GSH) levels, would be evaluated in future studies to provide a more comprehensive assessment of PRFSB’s antioxidant potential. Furthermore, since mitochondrial dysfunction is a key factor in SIM, investigating the effects of PRFSB on mitochondrial bioenergetics and apoptotic pathways would strengthen the mechanistic understanding of its protective actions.

Despite these limitations, this study provides compelling evidence for the protective role of PRFSB against atorvastatin-induced muscle toxicity. The findings support the potential use of PRFSB as a natural adjuvant in mitigating statin-associated adverse effects, which could improve patient adherence to lipid-lowering therapy.

## 5 Conclusion and future directions

This study highlights the significant myo-protective potential of the phenolic-rich fraction of *Solanum betaceum* (PRFSB) against atorvastatin-induced myotoxicity in female Wistar rats. By mitigating oxidative stress, reducing lipid peroxidation and normalizing muscle enzyme levels, PRFSB offers a promising adjunctive approach to alleviate the muscle-damaging effects of statins. The histopathological findings further corroborate these biochemical results, demonstrating preserved muscle architecture and reduced inflammation in treated animals. The polyphenol-rich composition of PRFSB, particularly its antioxidant properties, appears to play a critical role in its protective effects. These findings suggest that PRFSB could enhance the safety profile of statin therapy, providing a natural remedy for reducing the incidence of statin-induced muscle toxicity. Future research should explore additional mechanisms underlying the protective effects of PRFSB, particularly through the signaling of damage- or cell proliferation-related molecules. Techniques such as the TUNEL assay for apoptosis detection and RT-PCR or immunohistochemistry for validating specific markers of cell damage and proliferation, could provide valuable insights into the molecular pathways involved. Additionally, clinical studies will be crucial to evaluate the therapeutic potential of PRFSB in long-term statin therapy.

## CRediT authorship contribution statement

**Stella Maris Kembabazi:** Supervision. **Ibrahim Babangida Abubakar:** Methodology. **Ayomide Victor Atoki:** Writing – review & editing. **Nancy B. Mitaki:** Resources. **Josiah Eseoghene Ifie:** Supervision. **Ilemobayo Victor Fasogbon:** Software. **Rachael Jackie Mpumbya:** Writing – original draft, Project administration, Data curation. **Andrew Kisakye:** Validation. **Pastori Mujinya:** Software. **Siida Robert:** Investigation. **Fred Bwamble:** Methodology. **Mary Gorret Ablinda:** Resources. **Patrick Maduabuchi Aja:** Supervision. **Eliah Kwizera:** Software. **Solomon Adoni Mbina:** Investigation. **Daniel Ejike Eze:** Formal analysis. **Gilbert Akankwasa:** Formal analysis. **Sana Noreen:** Resources. **Nwokike Matthew Onyemaechi:** Resources.

## Declaration of Competing Interest

The authors declare that they have no known competing financial interests or personal relationships that could have appeared to influence the work reported in this paper.

## Data Availability

Data will be made available on request.

## References

[1] Acosta-quezada P.G., Raigón M.D., Riofrío-cuenca T., María D., Plazas M., Burneo J.I., Figueroa J.G., Prohens J. (2014). Diversity for chemical composition in a collection of different varietal types of tree tomato (*Solanum betaceum* Cav.), an Andean exotic fruit. FOOD Chem..

[2] Agu P.C., Aja P.M., Ekpono Ugbala E., Ogwoni H.A., Ezeh E.M., Oscar-Amobi P.C., Asuk Atamgba A., Ani O.G., Awoke J.N., Nwite F.E., Ukachi O.U., Orji O.U., Nweke P.C., Ekpono Ugbala E., Ewa G.O., Igwenyi I.O., Egwu C.O., Alum E.U., Chukwu D.C., Famurewa A.C. (2022). Cucumeropsis mannii seed oil (CMSO) attenuates alterations in testicular biochemistry and histology against Bisphenol a-induced toxicity in male Wister albino rats. Heliyon.

[3] Ahmed E.A., Abd-Eldayem A.M., Aboulhagag N.A. (2019). The possible protective effects of vitamin D and L-carnitine against atorvastatin-induced myopathy and hepatotoxicity. Comp. Clin. Pathol..

[4] Al-Kuraishy H.M., Al-Gareeb A.I., Al-Buhadily A.K. (2022). Histological assessment of tissue damage in experimental models of toxicity. J. Microsc. Ultrastruct..

[5] Anne Pascale K. (2019). Protective effect of ethanolic extract and raw juice of *Solanum betaceum* on aluminum-induced oxidative stress and associated memory deficits in rats. Investig. Med. Chem. Pharmacol..

[6] Bonifacio A., Sanvee G.M., Bouitbir J., Krähenbühl S. (2022). The oxidative stress hypothesis in statin-induced myotoxicity. Front. Pharmacol..

[7] Bouitbir J., Charles A.L., Rasseneur L., Dufour S., Piquard F., Geny B., Zoll J. (2019). Atorvastatin treatment reduces mitochondrial function and ATP content in human skeletal muscle cells: a potential mechanism underlying statin-induced myotoxicity. Arch. Toxicol..

[8] Collins R., Reith C., Emberson J., Armitage J., Baigent C., Blackwell L., Peto R. (2016). Interpretation of the evidence for the efficacy and safety of statin therapy. Lancet.

[9] Diep T., Pook C., Yoo M. (2020). Phenolic and anthocyanin compounds and antioxidant activity of tamarillo (*Solanum betaceum* Cav.). Antioxidants.

[10] Diep T.T., Rush E.C., Yoo M.J.Y. (2020). Tamarillo (*Solanum betaceum* Cav.): a review of physicochemical and bioactive properties and potential applications. Food Rev. Int..

[11] Fischer A.H., Jacobson K.A., Rose J.M. (2008). Hematoxylin and eosin staining: a review of techniques and their applications in histology. J. Histotechnol..

[12] Gonzalez-Covarrubias V., Beekman M., Uh H.W., Dane A., Troost J., Paliukhovich I., Hankemeier T. (2020). Lipidomics of statin-associated muscle symptoms: a pilot study. Eur. J. Clin. Pharmacol..

[13] Karahalil B., Hare E., Koç G., Uslu I., Şentürk K., Özkan Y. (2017). Hepatotoxicity associated with statins. Toksikol.

[14] Machado R.M., Marques-Aleixo I., Oliveira P.J., Ascensão A., Magalhães J. (2020). The role of mitochondria in statin-induced myotoxicity. Cells.

[15] Mahmoud A.R., Kamel O., Ahmed A. (2020). Alleviation of simvastatin-induced myopathy in rats by the standardized extract of Ginkgo biloba (EGb761): insights into the mechanisms of action. Cell Tissues Organs.

[16] Mancini G.B.J., Baker S., Bergeron J., Fitchett D., Frohlich J., Genest J., Gupta M., Hegele R.A., Ng D., Pearson G.J., Pope J., Tashakkor A.Y. (2016). Diagnosis, prevention, and management of statin adverse effects and intolerance: Canadian Consensus Working Group Update (2016). Can. J. Cardiol..

[17] OECD. (2022). Test Guideline 425: Acute Oral Toxicity - Up-and-Down Procedure. Guideline for Testing of Chemicals, 4(December), 26. https://doi.org/https://doi.org/10.1787/9789264071049-en.

[18] Rito M., Marques J., da Costa R.M.F., Correia S., Lopes T., Martin D., Canhoto J.M.P.L., Batista de Carvalho L.A.E., Marques M.P.M. (2023). Antioxidant potential of tamarillo fruits—Chemical and infrared spectroscopy analysis. Antioxidants.

[19] Rohilla A., Mahanta D. (2021). Bioguided isolation and antioxidant evaluation of phenolic-rich fractions from Terminalia chebula Retz. fruits. J. Food Biochem..

[20] Rosenson R.S., Baker S.K., Jacobson T.A., Kopecky S.L., Parker B.A. (2019). An assessment by the Statin Muscle Safety Task Force: 2019 update. J. Clin. Lipidol..

[21] Saleem U., Amin S., Ahmad B., Azeem H., Anwar F., Mary S. (2017). Acute oral toxicity evaluation of aqueous ethanolic extract of Saccharum munja Roxb. roots in albino mice as per OECD 425 TG. Toxicol. Rep..

[22] Singh R., Chaturvedi P. (2019). Phytochemical Characterization of Rhizome, Fruit, Leaf and Callus of Rheum emodi Wall. using GC-MS. Pharmacogn. J..

[23] Upadhyay R., Mohan Rao L.J. (2019). Antioxidant and anti-inflammatory activities of chlorogenic acid: a comprehensive review. Phytother. Res..

[24] Usman L.A., Ali H.M., Al-Dosary M.K. (2016). Histological and biochemical study on the effects of herbal extracts in reducing oxidative stress. J. Histol. Histopathol..

[25] Vauzour D., Rodriguez-Mateos A., Corona G., Oruna-Concha M.J., Spencer J.P.E. (2018). Polyphenols and human health: prevention of disease and mechanisms of action. Nutrients.

[26] Vina J., Borras C., Miquel J. (2018). The role of estrogen in the regulation of mitochondrial function and the prevention of oxidative damage. Ann. N. Y. Acad. Sci..

[27] Wang S., Zhu F. (2019). Tamarillo (*Solanum betaceum*): chemical composition, biological properties, and product innovation. Trends Food Sci. Technol..

[28] Welz A.N., Emberger-Klein A., Menrad K. (2018). Why people use herbal medicine: insights from a focus-group study in Germany. BMC Complement. Altern. Med..

[29] Zanatta C.F., Boff T.A., Martins J.S., Weirich M., Alves A.O., Leal Junior W., Alves M.A. (2020). Characterization of polyphenolic compounds in tamarillo (Solanum betaceum) and its antioxidant and anti-inflammatory activities. Food Res. Int..

[30] Zhang Y., Ma X., Li H., Zhang H., Kong L. (2021). Phytochemicals as potential therapeutics for statin-induced myopathy: targeting mitochondrial dysfunction and oxidative stress. Crit. Rev. Food Sci. Nutr..

[31] Zhang Y., Yang S., Li X., Liu J., Wang L. (2021). Identification of bioactive compounds in medicinal plants and their potential mechanisms for modulating oxidative stress, lipid metabolism, and inflammation. Phytochem. Rev..

